# 25 Years of Collaboration with A Genius: Deciphering Adenine Nucleotide Ca^2+^ Mobilizing Second Messengers Together with Professor Barry Potter

**DOI:** 10.3390/molecules25184220

**Published:** 2020-09-15

**Authors:** Andreas H. Guse

**Affiliations:** The Calcium Signalling Group, Dept. of Biochemistry and Molecular Cell Biology, University Medical Center Hamburg-Eppendorf, Martinistrasse 52, D-20246 Hamburg, Germany; guse@uke.de; Tel.: +49-40-7410-52828

**Keywords:** adenine nucleotide, second messenger, cADPR, NAADP, ADPR, 2′-deoxy-ADPR, Ca^2+^ signaling, T cell activation

## Abstract

Ca^2+^-mobilizing adenine nucleotide second messengers cyclic adenosine diphosphoribose, (cADPR), nicotinic acid adenine dinucleotide phosphate (NAADP), adenosine diphosphoribose (ADPR), and 2′deoxy-ADPR were discovered since the late 1980s. They either release Ca^2+^ from endogenous Ca^2+^ stores, e.g., endoplasmic reticulum or acidic organelles, or evoke Ca^2+^ entry by directly activating a Ca^2+^ channel in the plasma membrane. For 25 years, Professor Barry Potter has been one of the major medicinal chemists in this topical area, designing and contributing numerous analogues to develop structure–activity relationships (SAR) as a basis for tool development in biochemistry and cell biology and for lead development in proof-of-concept studies in disease models. With this review, I wish to acknowledge our 25-year-long collaboration on Ca^2+^-mobilizing adenine nucleotide second messengers as a major part of Professor Potter’s scientific lifetime achievements on the occasion of his retirement in 2020.

## 1. Introduction

A ‘genius’ is defined as, “very great and rare natural ability or skill, especially in a particular area such as science or art, or a person who has this” [1]; truly Professor Barry Potter fulfills this definition. Having utmost great skills in molecular life sciences combined with the rare ability to drive the topical field of second messengers, over the past four (!) decades he shaped this area of life science.

“Ca^2+^ signaling controlled by Ca^2+^-mobilizing adenine nucleotide second messengers—that’s a cool innovative idea that should be pursued—it just needs an interesting cell system and novel tools developed by medicinal chemistry”, Barry Potter said while having lunch in a small Italian–Swiss restaurant in Hamburg in fall of 1994. Indeed, our over-lunch conversation turned out to be the starting point of a very fruitful 25-year-long collaboration with the first paper, showing Ca^2+^ release by cyclic adenosine diphosphoribose (cADPR) in T-lymphocytes, published in 1995 [2]. Interestingly, cADPR was not the only Ca^2+^-mobilizing adenine nucleotide second messenger discovered in the 1990s: another even more potent compound, nicotinic acid adenine dinucleotide phosphate (NAADP), was reported in 1995 by Professor Hon Cheung Lee’s laboratory [3]. A further Ca^2+^-mobilizing adenine nucleotide second messenger, adenosine diphosphoribose (ADPR), discovered as an agonist of the cation channel transient receptor potential, melastatin subfamily type 2 (TRPM2; [4]), then became the third major bond of our collaboration.

For all these endogenous messengers, our project goal was to develop structure–activity relationships (SAR) as a basis for tool development for biochemistry and cell biology and for lead development in proof-of-concept studies in disease models, such as experimental autoimmune encephalomyelitis (EAE).

Without being able to fully estimate what our collaboration has meant to Barry’s career, I can say that it influenced my own scientific curriculum significantly. Therefore, this review is not meant to systematically cover the world of Ca^2+^-mobilizing adenine nucleotide second messengers, but I wish to highlight the milestone studies developed and published in collaboration with Barry.

Since the discovery of d-*myo*-inositol 1,4,5-trisphosphate (IP_3_) as a principal second messenger evoking Ca^2+^ release from intracellular stores in 1983 [5], the term Ca^2+^ signaling has been filled with more and more scientific results and conclusive concepts. This was the case, since rapidly the IP_3_ signaling pathway was defined by the discovery of the key proteins involved: phospholipase C, IP_3_ kinase and IP_3_ phosphatase, as well as the IP_3_ receptor(s). Further, IP_3_, as a signal to connect the stimulation of plasma membrane (PM) receptors with intracellular Ca^2+^ signaling, fueled research regarding other small molecules having similar functions in cells.

Indeed, cADPR and NAADP were soon discovered and structurally defined [3,6], followed somewhat later by ADPR [4]. Since all these messengers are metabolic products of nicotinamide adenine dinucleotide (NAD), known at that time mainly as substrates of NAD-dependent oxidoreductases, they can be summarized as members of the superfamily of adenine nucleotide second messengers.

## 2. Stages of T Cell Activation and T Cell Ca^2+^ Signaling

**Figure 1:**
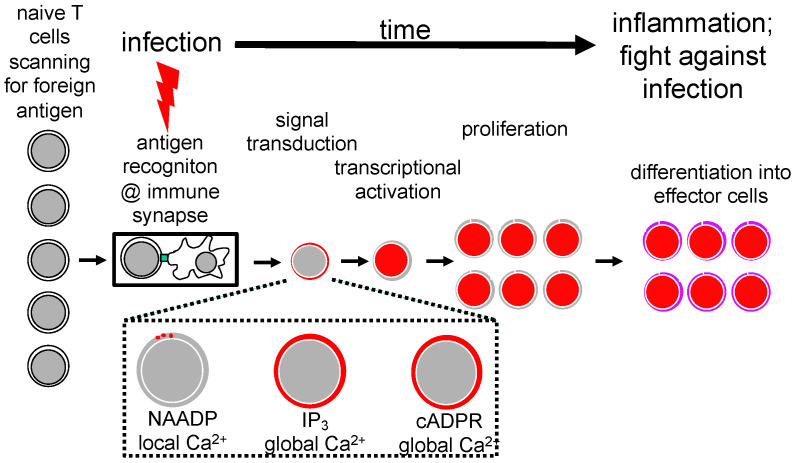
Ca^2+^ signaling as an essential part of signal transduction during T cell activation in infection. Figure 1 displays schematically the start of activation of T cells by APC in the context of the immune synapse and the subsequent steps of signal transduction, including Ca^2+^ signaling, transcriptional activation, proliferation and finally differentiation to effector T cells that ultimately fight infection.

T cells are activated during an infection by antigenic peptides presented by antigen-presenting cells (APC), such as dendritic cells, macrophages or B-lymphocytes. In auto-immunity, auto-antigenic peptides replace the foreign peptides. The term ‘T cell activation’ relates to all biochemical and cell biology processes following the formation of a juxtacrine intimate contact between an APC and the T cell, termed the immunological synapse or immune synapse. Upon immune synapse formation, we can distinguish between different subsequent stages of T cell activation (Figure 1). Signal transduction comprises Ca^2+^ signaling, besides other signaling pathways (Figure 1). Ultimately, T cell transcription is activated and T cells are driven into proliferation and finally differentiate into effector T cells that fight infection (Figure 1). 

Ca^2+^ signaling can be further subdivided into NAADP- and ORAI1-dependent local Ca^2+^ signals [7,8], IP_3_-dependent global Ca^2+^ signaling, and cADPR and ryanodine receptor (RYR)-dependent global Ca^2+^ signaling (Figure 1). In the following sections, I will first concentrate on NAADP and cADPR as two of the three main bonds between Barry Potter’s lab and my group.

## 3. Nicotinic Acid Adenine Dinucleotide Phosphate (NAADP)

**Figure 2:**
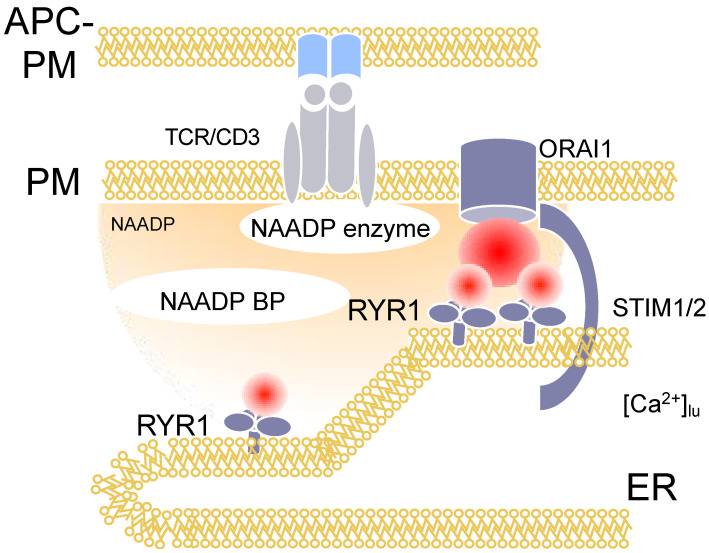
Ca^2+^ signaling in the first 15 s of T cell activation: NAADP-dependent Ca^2+^ microdomains. Figure 2 displays schematically the events within the first 15s of activation of T cells in or close to ER-plasma membrane junctions. NAADP is formed by a so far unknown enzyme, then binds to an NAADP binding protein, which, bound to its ligand, then activates local Ca^2+^ release via RYR1.

The first signals that can be monitored during T cell activation are local, short-lived and highly dynamic Ca^2+^ signals, termed Ca^2+^ microdomains [7,8]. Besides gene silencing approaches to disentangle the molecular mechanisms involved, an analogue of NAADP synthesized initially by Barry Potter’s group was used, namely BZ194 [9]. The hypothesis underlying the design of BZ194 was that the nicotinic acid moiety of NAADP is the most relevant group for its Ca^2+^ mobilizing activity. We confirmed this by microinjection experiments where nicotinic acid abolished Ca^2+^ signaling evoked by NAADP [9]. Then, nicotinic acid was fused to different types of lipophilic groups and screened in a rat T cell proliferation assay. One of the hits, BZ194, was then validated by microinjection experiments as above and resulted in an IC_50_ of approximately 5 μM towards NAADP evoked Ca^2+^ signaling; furthermore, when BZ194 was co-injected with IP_3_ or cADPR, no inhibition was observed, demonstrating the specificity of this NAADP antagonist [9]. BZ194 significantly decreases the number of Ca^2+^ microdomains within the first 15 s of T cell activation in primary T cells [8]. Mechanistically, BZ194 interferes with RYR1 opening, as shown in [^3^H]ryanodine binding assays [9]. However, the current model for NAADP signaling includes a molecularly unidentified binding protein for NAADP (Figure 2: NAADP-BP), which mediates the interaction between NAADP and RYR1. NAADP BP was characterized by photoaffinity labeling as a small cytosolic protein in T cells [10].

The significant drop of initial Ca^2+^ microdomain numbers upon NAADP antagonism by BZ194 also significantly affected global Ca^2+^ signaling in primary T cells in a characteristic manner: (i) signal onset was delayed, and both peak and plateau amplitudes of Ca^2+^ signaling were decreased partially [9]. This result is interpreted mechanistically as a lack of co-activation of both IP_3_Rs and RYRs by local Ca^2+^ increases evoked normally by NAADP. Consistently, downstream T cell activation events, such as nuclear translocation of NFAT-1 or IL-2 expression, were also partially inhibited by BZ194. Importantly, the partial inhibition of proliferation by BZ194 was bypassed using a low concentration of ionomycin together with anti-CD3/anti-CD28 monoclonal antibodies. Here, the missing local, NAADP-evoked Ca^2+^ signals were restored by Ca^2+^ ionophore ionomycin, resulting in full proliferation [8].

BZ194 was then used to characterize the role of NAADP-evoked Ca^2+^ signaling in T cell-mediated autoimmunity of the central nervous system (CNS) [11]. NAADP antagonist BZ194 decreased T cell motility from the spleen to the CNS. Further, re-activation of effector T cells upon arrival in the nervous tissues was attenuated, resulting in strongly reduced levels of the pro-inflammatory cytokines interferon-gamma and interleukin-17. In the animal model disease experimental autoimmune encephalomyelitis (EAE), a model for multiple sclerosis, the clinical symptoms were ameliorated. BZ194 induced a transient state of non-responsiveness, which was observed in post-activated effector T cells only, but not in naïve or memory T cells. Interestingly, RYR1 expression was increased in effector T cells as compared to naïve or memory T cells [11].

Taken together, the development of NAADP antagonist BZ194 was a major step towards mechanistically understanding the role of NAADP in both local and global Ca^2+^ signaling in T cells (Figure 2), as well as for T cell biology during autoimmunity of the CNS.

The use of NAADP antagonist BZ194 is not limited to T cells. We demonstrated the involvement of NAADP-evoked Ca^2+^ signaling in spontaneous diastolic Ca^2+^ transients in cardiac myocytes stimulated via the β1-adrenoceptor [12], and, more recently, a contribution of NAADP to glutamate-evoked Ca^2+^ signaling in mouse hippocampal neurons [13].

## 4. Cyclic Adenosine Diphosphoribose (cADPR)

As introduced above, IP_3_ is one of the second messengers involved in T cell Ca^2+^ signaling. Its kinetics are slower as compared to NAADP, with a peak at around 3 min and a full return to baseline values within approximately 15 min [14]. This result was found in mass measurements of IP_3_ using anion-exchange HPLC coupled to post-column complexometry (metal-dye detection HPLC) [15]. Then, the question arose as to how store-operated Ca^2+^ entry (SOCE) can continue in the absence of IP_3_-evoked Ca^2+^ release, since SOCE is mechanistically triggered by a decrease in the luminal ER-Ca^2+^ concentration, usually resulting from second messenger-evoked Ca^2+^ release [16].

**Figure 3:**
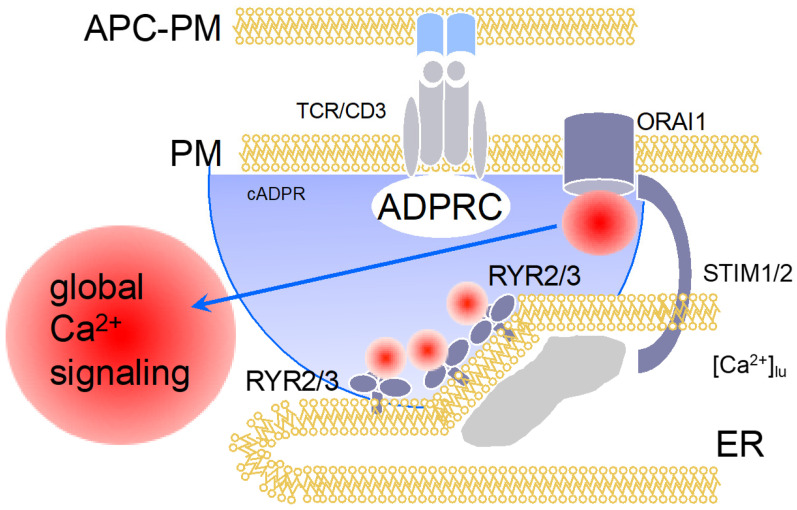
Ca^2+^ signaling in a later phase (>15 min) of T cell activation: cADPR-dependent global Ca^2+^ signaling. Figure 3 displays schematically the events at >15 min of T cell activation. cADPR is formed, either by CD38 or by a so far unknown enzyme, then activates local Ca^2+^ release via RYR12 and/or RYR3, resulting in substantially diminished luminal ER-Ca^2+^ concentration (shown by the grey area below the ER membrane) that keeps ORAI1 open via SOCE.

The answer to this important question of T cell biology was tackled by Barry Potter’s group and my group by hypothesizing that cADPR might be the 2nd messenger taking over the role of IP_3_ at later time points of T cell activation, e.g., approximately >15 min post stimulation. For this, we characterized cADPR-evoked Ca^2+^ release in permeabilized T cells [2,17], and showed for the first time that cADPR activates Ca^2+^ entry in intact T cells [18]. The latter result was interpreted as cADPR-evoked Ca^2+^ release that subsequently activated SOCE [18]. Then, a 2-step HPLC method for quantification of cADPR was developed [19] and revealed relatively slow kinetics that reached a top within 5 min post stimulation and stayed level for at least the first hour post stimulation [20]. Further, using a stable and efficient cADPR antagonist developed by Barry Potter and his team, 7-deaza-8-Br-cADPR [21], we demonstrated in Ca^2+^ imaging experiments a major role for cADPR in sustained Ca^2+^ entry, in the expression of T cell activation markers, and in T cell proliferation [20]. This role of cADPR for this ‘late’ phase of Ca^2+^ entry was also demonstrated by combined microinjection and Ca^2+^ imaging experiments with antagonists developed and synthesized by Barry, Potter, e.g., cADPR antagonist 8-OCH_3_-cADPR and IP_3_ antagonist inositol 1,4,6-phosphorothioate [20]. Another important result of this study was the demonstration of expression of RYR in T cells, a proven fact that had been questioned a few years before [22]. Taken together, cADPR, by evoking Ca^2+^ release via RYR2 and/or RYR3, activates SOCE in T cells (Figure 3).

**Figure 4:**
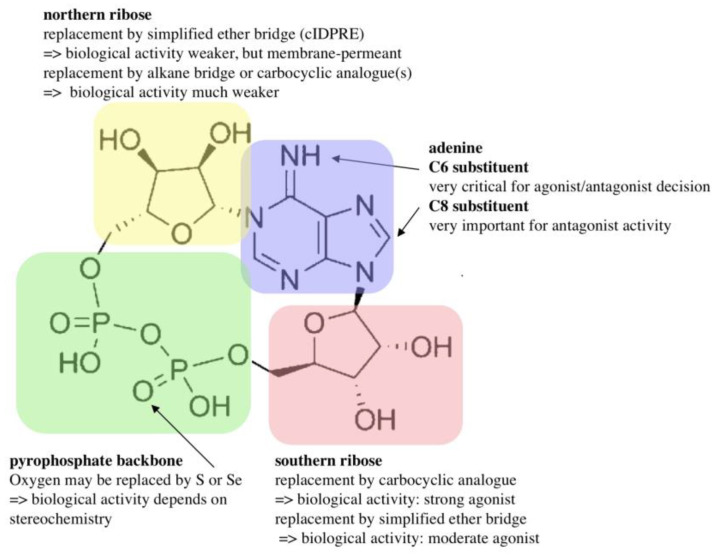
Structure–activity relationship of cADPR.

Subsequently, the Potter lab designed and synthesized numerous cADPR analogues to complete the structure–activity relationship. Many of those were also tested in T cells, e.g., 2”-NH_2_-cADPR [23], cyclic aristeromycin diphosphoribose [24], and *N*1-cyclic inosine diphosphoribose and 8-modified analogues thereof [25,26,27,28,29]. While the carbocyclic derivative of the northern ribose of cADPR was only characterized as a weak agonist in T cells [30], the thioribose analogue, cADP-4-thio-ribose (cADPtR), proved to be a full agonist [31,32]. A more comprehensive review of the structure–activity relationship of cADPR is shown in Figure 4 [33].

Collaborating on the cADPR structure–activity relationship with Barry Potter was an ultimate pleasure. Not only did he make numerous analogues, many of which proved to be very helpful to decipher T cell Ca^2+^ signaling (see paragraphs above); moreover, he convinced brilliant chemists, like Professor Satoshi Shuto (Hokkaido University, Japan), who spent time as a guest scientist in Barry’s lab at the University of Bath, to make further complicated analogues, e.g., the carbocylic and thioribose derivatives at the northern ribose of cADPR.

## 5. Adenosine Diphospho-Ribose (ADPR) and 2′-Deoxy-ADPR (2dADPR)

As mentioned in the first paragraph, besides NAADP and cADPR, another endogenous adenine nucleotide was shown to be a second messenger in Ca^2+^ signaling, the molecule ADPR. Initially, ADPR was simply seen as degradation product of NAD and cADPR, formed by NAD-glycohydrolase/ADP-ribosyl cyclase CD38. However, Peraud et al. demonstrated in 2001 that TRPM2 contained a domain with homology to the nudix family of nucleotide hydrolases, termed the NUDT9-homology domain [4]. The NUDT9-homology domain binds ADPR and this ligand binding increases the open probability of TRPM2 [4].

In 2008/09, Barry Potter, his co-workers, and members of my lab discussed synthesis of ADPR analogues for a full structure–activity relationship of ADPR. In a systematic approach, the synthesis of > 30 ADPR derivatives with modifications in each of the four motifs, (i) adenine base, (ii) adenosine ribose, (iii) pyrophosphate bridge, and (iv) terminal ribose, was carried out [34]. Using recombinant TRPM2 stably expressed in HEK293 cells, ADPR analogues were infused via patch pipette in whole-cell mode, and K^+^ outward currents at +15 mV were used to quantify antagonism of ADPR-evoked TRPM2 activation [34]. ADPR analogues with a purine C8 substituent showed antagonist activity and the 8-phenyl substitution was very effective with an IC_50_ of approximately 10 μM [34]. This antagonist activity was significantly increased by a hybrid structure, 8-phenyl-2′-deoxy-ADPR (IC_50_ = 3 μM) [34]. Modifications in the other moieties of ADPR either resulted in weak antagonists or antagonist activity was abolished, e.g., in bioisosteric replacement of the pyrophosphate linkage [34].

**Figure 5:**
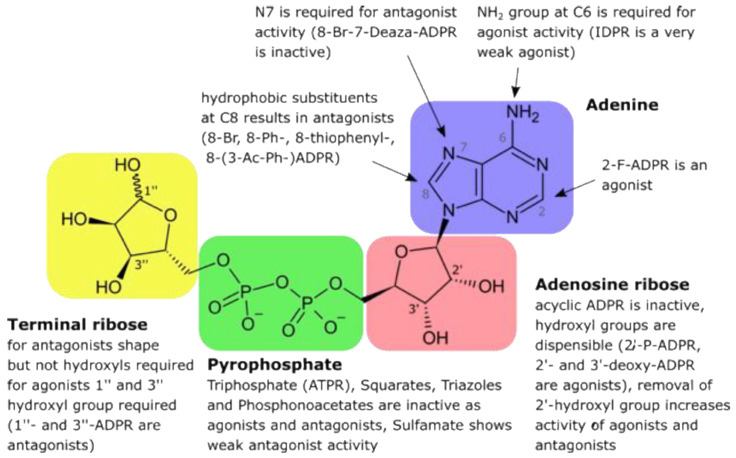
Structure–activity relationship of ADPR.

Then, an almost identical set of ADPR analogues was used in the same experimental setting to explore the agonist structure–activity relationship of ADPR [35]. Since relatively high concentrations of ADPR had been shown to be necessary for TRPM2 activation, e.g., ≥ 100 μM ADPR, indicating a low affinity interaction between the ligand and TRPM2, Barry and I expected that in the worst case many of the ADPR analogues might activate TRPM2. However, this turned out not to be the case: among the various ADPR analogues, only 2′-deoxy-ADPR, 3′-deoxy-ADPR, 2′-phospho-ADPR, and 2-F-ADPR were agonists of TRPM2 [35]. More excitingly, 2′-deoxy-ADPR was a significantly better TRPM2 agonist as compared to ADPR that evoked 10.4-fold higher whole cell currents at saturation. Both a higher average open probability and a decreased rate of inactivation of TRPM2 were found to be the molecular determinants of the super agonist activity of 2′-deoxy-ADPR [34]. The structure–activity relationship of ADPR is summarized in Figure 5.

A second aspect of this study was the possible 2nd messenger role of 2′-deoxy-ADPR: using HPLC and mass spectrometry, endogenous 2′-deoxy-ADPR was not only detected in unstimulated Jurkat T-lymphocytes, but a T cell receptor/CD3-mediated increase in 2′-deoxy-ADPR was discovered for the first time [35]. Here, Barry and his team were also very important since they succeeded in determining the molecular ion of endogenous 2′-deoxy-ADPR from extracts of T cells. Finally, the lack of detection of 2′-deoxy-ADPR in extracts from *Cd38*^−/−^ T cells suggest the formation of this molecule by NAD-glycohydrolase activity of CD38 [35].

Further studies on the structure–activity relationship of ADPR followed, showing a critical role for OH-groups at C1”, C2” and C3” at the terminal ribose for agonist properties of ADPR [36] and demonstrating again that replacement of the pyrophosphate bridge, here replacement by a phosphonoacetate linker, resulted in the loss of any biological or pharmacological activity [37].

## 6. Conclusions

In a true scientific collaboration, Barry Potter, myself and both our teams drastically changed the view of T cell Ca^2+^ signaling. While IP_3_ followed by IP_3_-initiated/mediated activation of SOCE was regarded as the whole story in the 1990s, we added both NAADP and cADPR signaling as essential components of T cell activation. Further, we discovered 2′-deoxy-ADPR as a novel 2nd messenger activating TRPM2 much more effectively than ADPR.

Looking back to the past 25 years with the many personal interactions Barry and I had, either in Bath, Hamburg or during scientific meetings and conferences, I am delighted to say that he has been one of the most important collaborators I ever had during my scientific career.

I well remember one of our last personal meetings during the International Association for Cellular Coenzymes (IACC) Inauguration Symposium (July 2018 at UCL, London) at Dillon’s Coffee located at the corner of Gower Street/Torrington Place in summer 2018. Sitting outside in one of the congress breaks in bright sunshine with a cappuccino, Barry said: “Really fortunate that we met and started our collaboration many years ago…with excellent results, but more important with a lot of fun and friendship!”
